# Analysis of oxidase activity and transcriptomic changes related to cutting propagation of hybrid larch

**DOI:** 10.1038/s41598-023-27779-x

**Published:** 2023-01-24

**Authors:** Ruofan Qin, Qingrong Zhao, Chenrui Gu, Chen Wang, Lei Zhang, Hanguo Zhang

**Affiliations:** State Key Laboratory of Tree Genetics and Breeding (Northeast Forestry University), Harbin, 150040 China

**Keywords:** Plant breeding, Plant development, Plant genetics, Plant hormones, Plant molecular biology, Plant physiology

## Abstract

Hybrid larch is the main timber and afforestation tree species in Northeast China. To solve the problem of rooting difficulties in larch cutting propagation, enzyme activity determination and transcriptome sequencing were carried out on the rooting tissues at five timepoints after cutting. peroxidase (POD), indole acetic acid oxidase (IAAO) and polyphenol oxidase (PPO) play important roles in the larch rooting process after cutting. A total of 101.20 Gb of clean data was obtained by transcriptome sequencing, and 43,246 unigenes were obtained after further screening and assembly. According to GO analysis and KEGG enrichment analysis, we think that plant hormones play an important role in the rooting process of larch stem cuttings. in the plant hormone signal transduction pathway, a larch gene c141104.graph_c0 that is homologous to the Arabidopsis *AUX1* was found to be significantly up-regulated. We suggest that *AUX1* may promote IAA transport in larch, thus affecting adventitious root development. According to the results of POD, PPO IAAO indexes and GO analysis, we think s1 and s2 periods may be important periods in the rooting process of larch stem cuttings, so we built a gene regulatory network, a total of 14genes, including *LBD*, *NAC*, *AP2/ERF*, *bHLH* and etc., may be important in different stages of cutting propagation. As the rooting rate after cutting inhibits the development of larch clone propagation, identifying the genes that regulate rooting could help us to preliminarily understand the molecular mechanism of adventitious root formation and select a better treatment method for cutting propagation.

## Introduction

Larch is an important coniferous tree species in Northeast China that has the characteristics of early rapid growth and strong durability, so it is a good timber species for forest production^1^. Vegetative or clonal propagation in forest tree breeding has the advantages of convenient operation, a short breeding cycle and high wood yield per unit area. However, some groups, including economically important species, are difficult to root. The regulation of rooting stage is affected by a large number of factors, among which plant hormones, polyamines and oxidases seem to be factors affecting and regulating adventitious root (AR) formation^2^. Larch is a difficult-rooting tree species, and the low rooting rate of cuttings restricts the use of cutting propagation to popularize larch clones.

Cutting propagation of plants is a complex physiological activity that not only requires nutrients as substrates to supply energy but also requires photosynthesis and respiration^3^. Therefore, a variety of enzymes in plants are involved in the development of ARs. Many studies have shown that POD, IAAO and PPO are closely related to the occurrence of ARs and the morphogenesis of plant organs.

The formation of ARs is affected by genetic and environmental factors, in which plant hormones play a major role^4^. Auxins, especially indole-3-butyric acid (IBA) might enhance the signal of auxin directing to founder cells for AR initiation^5^. Ethylene can play a role in the development of ARs by regulating the metabolism of auxin. Gibberellin promotes the formation of ARs and lateral roots to a certain extent; furthermore, an appropriate concentration of cytokinin is also an important regulator of AR formation in stem segments^6^. Other hormones, such as jasmonic acid (JA), have been shown to have phase-dependent effects, inhibiting early induction but promoting root formation^7^. Auxin early response genes mainly include three types: *Aux/IAA*, *SAUR*, and *GH3*. Studies have shown that the protein encoded by the *Aux/IAA* gene mainly acts on the auxin signal transduction pathway; when auxin binds to its receptor, it activates some transcription factors in the nucleus to promote gene expression^8^. The *ARF* gene (auxin response factor) family, which interacts with the *Aux/IAA* gene family, is another auxin-related gene that has been widely studied. *ARF* proteins are transcription factors that bind closely to auxin response elements. These auxin response elements target the promoters of many auxin-related genes^9^.

At present, with the development and application of gene chip and RNA-Seq technology, research on plant molecular biology has been greatly promoted. Great progress has also been made in the study of the molecular mechanism of plant root development^10–13^. Han et al.^14^ used two clones of *L. kaempferi* × *L. olgensis*, 25–5 (strong rooting capacity) and 23–12 (weak rooting capacity), as their research materials. They performed 454 sequencing technology analysis of the transcriptome data during the formation of ARs of hybrid larch, obtained 45,137 contigs and 61,647 singletons. A joint analysis of transcriptome and proteome showed that genes related to two pathways, polyamine synthesis and stress response, might play an important role in AR development. Shang et al.^15^ used hardwood cuttings of mulberry to explore the transcriptome changes of early AR formation. The results showed that auxin had a positive effect on AR induction and revealed the key role of hormone signal transduction, stress response and circadian rhythm in coordinating the early physiological changes during AR formation in mulberry cuttings. To deeply and accurately mine and identify genes related to the rooting of larch during cutting propagation and find the main signalling pathways involved, we used larch stem cuttings as materials to construct cDNA libraries for five important stages of adventitious root development. We also analysed the transcriptome data produced through high-throughput sequencing to obtain genes related to AR formation in larch and reveal its rooting mechanism.

## Results and analysis

### Oxidase activity analysis during rooting periods after cutting propagation

**Figure 1:**
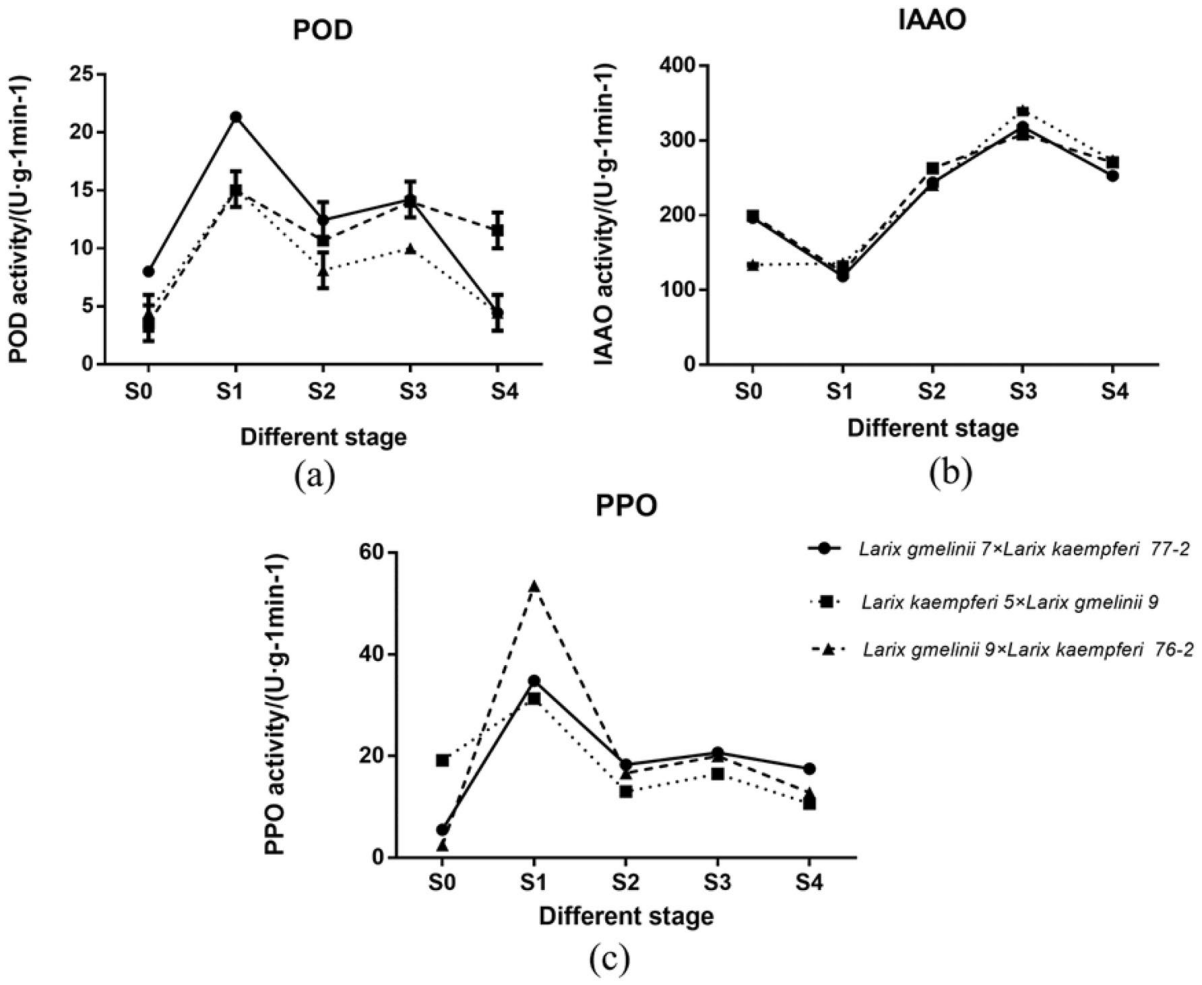
POD, IAAO and PPO activities of three larch clones at five stages of adventitious root formation.

POD is a kind of peroxidase with high activity which widely exists in plants, plays an important role in cell differentiation and has high activity. At the initial stage of cutting (Fig. 1), the POD activity of the cuttings increased rapidly to the highest level. We speculate that POD was beneficial to the formation of calli and ARs and that it could synthesize root-promoting substances, which is beneficial to the rooting of cuttings. IAAO can decompose IAA and regulate the level of IAA in plants. Thus, the activity of the IAAO enzyme affects the formation of cutting root primordia. The reduction of IAAO activity at the beginning of cutting is helpful to reduce IAA decomposition in plants, which is beneficial to the rooting of cuttings. After AR formation, IAA synthesis decreased, and IAAO activity increased. At the initial stage of cutting, the activity of PPO reached the maximum and decreased gradually with the induction of ARs; therefore, the activity of PPO may be related to the emergence of calli. The dynamic changes in POD, IAAO and PPO in the three clones were consistent, and the magnitude of the changes in the s1 period was proportional to the degree of promotion of AR formation.

### Analysis of sequencing and assembly results

A total of 101.20 Gb of clean data was obtained from the library composed of 15 larch samples, and the clean data for each sample was 5.74 Gb, with the percentage of Q30 bases being 93.75% and above. A total of 43,246 unigenes were obtained after assembly; the N50 values of the transcripts and unigenes were 1891 and 1799, respectively, and the integrity of the assembly was high. There were 17,220 unigenes with lengths greater than 1 kb.

### Functional annotation and classification of differentially expressed genes

**Figure 2:**
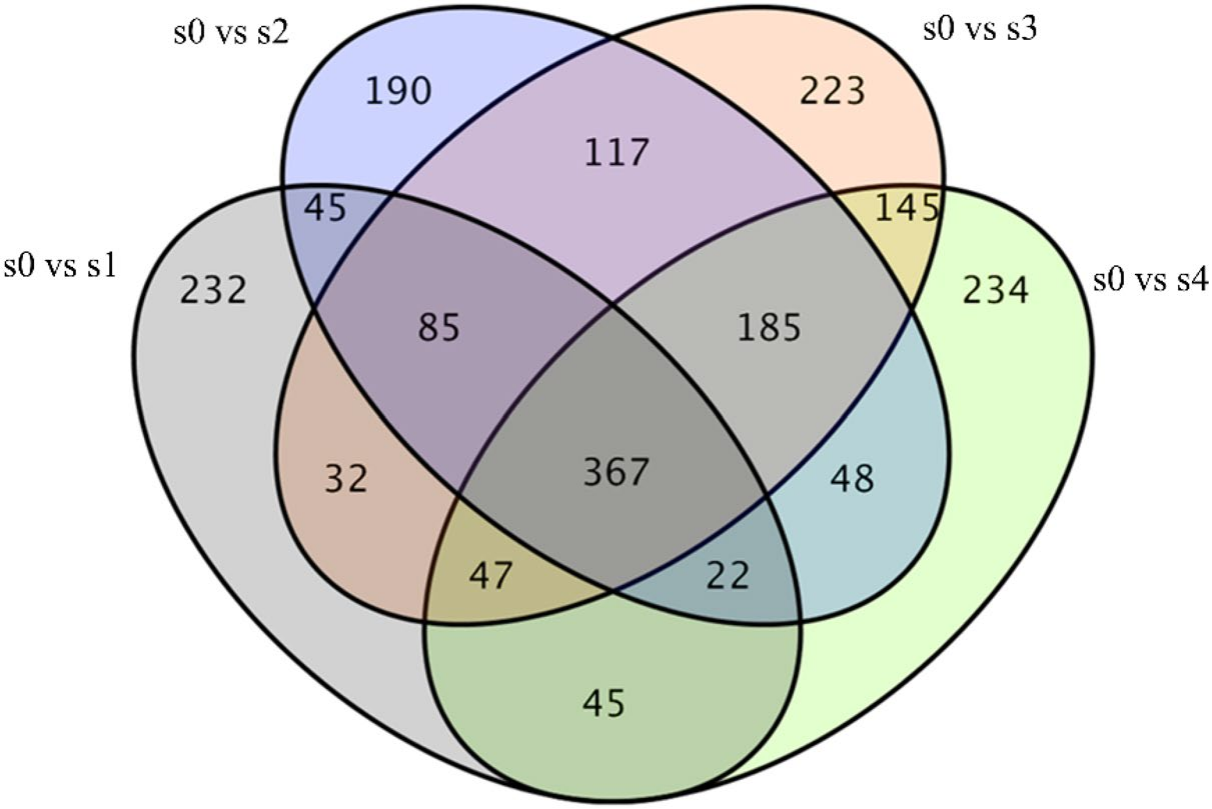
Venn diagram of DEGs.

The BLAST parameter E-value ≤ 1e−5 and the HMMER parameter E-value ≤ 1e−10 were selected. Finally, 31,234 unigenes with annotation information were obtained, and the most annotated databases were NR (29,944, 95.87%), GO (23,210, 74.31%) and Pfam (22,221, 71.14%). The genes obtained from sequencing were analyzed using FDR < 0.01 and FC > 2 as the filtering criteria, and the number of differentially expressed genes obtained was shown in Table 1. In addition, according to the Venn diagram (Fig. 2), 367 differentially expressed genes were detected at all four stages of adventitious root development.

**Table 1:** Statistics of number of differentially expressed genes.

DEG set	All DEG	Up-regulated	Down-regulated
s0 vs s1	2220	875	1345
s0 vs s2	2448	1059	1389
s0 vs s3	2887	1201	1686
s0 vs s4	2328	1093	1235

### Enrichment analysis of differentially expressed genes by gene ontology (GO)

**Figure 3:**
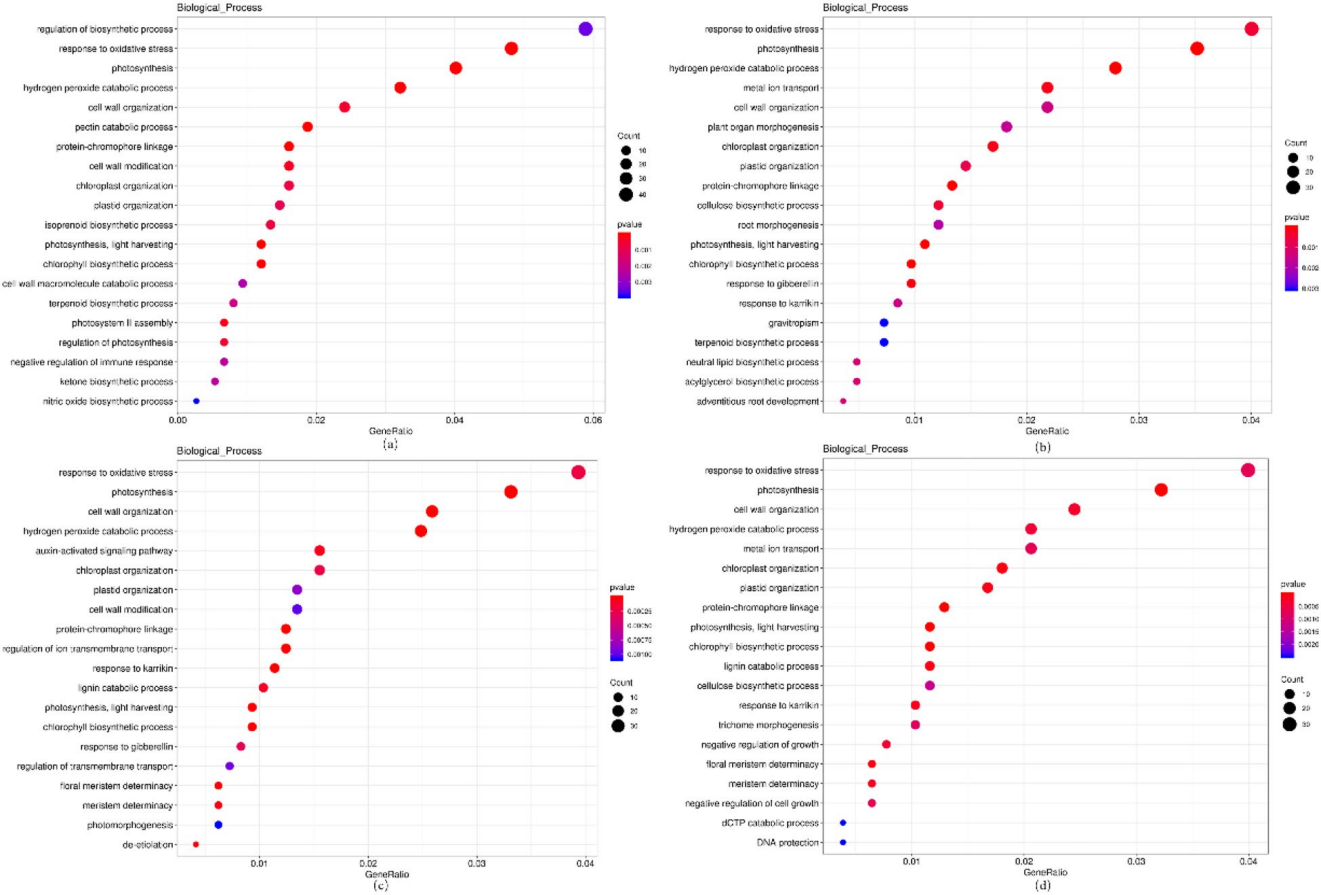
Statistics of the GO annotation classification for the DEGs. (**a**) s0 vs. s1; (**b**) s0 vs. s2; (**c**) s0 vs. s3; (**d**) s0 vs. s4. The abscissa is the GO classification, the right side is the number of genes. From the picture above, we can see the annotation of DEGs and all genes in the secondary GO categories.

With p-value < 0.05 as the threshold, GO Term is defined as an item significantly enriched in differentially expressed genes. There are three categories in the Gene Ontology database: Molecular Function, Cellular Component and Biological Process, Biological process is the key module of this study As shown in the figures, genes in s0 vs s1 stage were mainly enriched in the post-embryonic plant morphogenesis (GO:0090698), post-embryonic plant organ development (GO:0090696),post-embryonic root morphogenesis (GO:0010101), root morphogenesis (GO:0010015), lateral root morphogenesis (GO:0010102); The genes in s0 vs s2 stage were mainly enriched in the plant organ morphogenesis (GO:1905392), root morphogenesis (GO:0010015), gravitropism (GO:0009630), adventitious root development (GO:0048830); There was no significant enrichment for pathways with a clear relationship to rooting from s3, but some plant hormone related pathways such as auxin-activated signaling pathway (GO:0009734), response to gibberellin (GO:0009739) had been enriched. Therefore, we speculated that embryogenic callus was formed in s1, in this stage root morphogenesis was just started. Root morphogenesis changed to adventitious root development in s2, and in s3, the accumulation of plant hormones and other substances induced the elongation and growth of pre-formed adventitious roots. There was no enrichment pathway related to root development in s4, indicated that the important stage of root growth and development had been completed and normal plant growth had entered (Fig. 3).

### Kyoto encyclopedia of genes and genomes (KEGG) analysis of the DEGs

**Figure 4:**
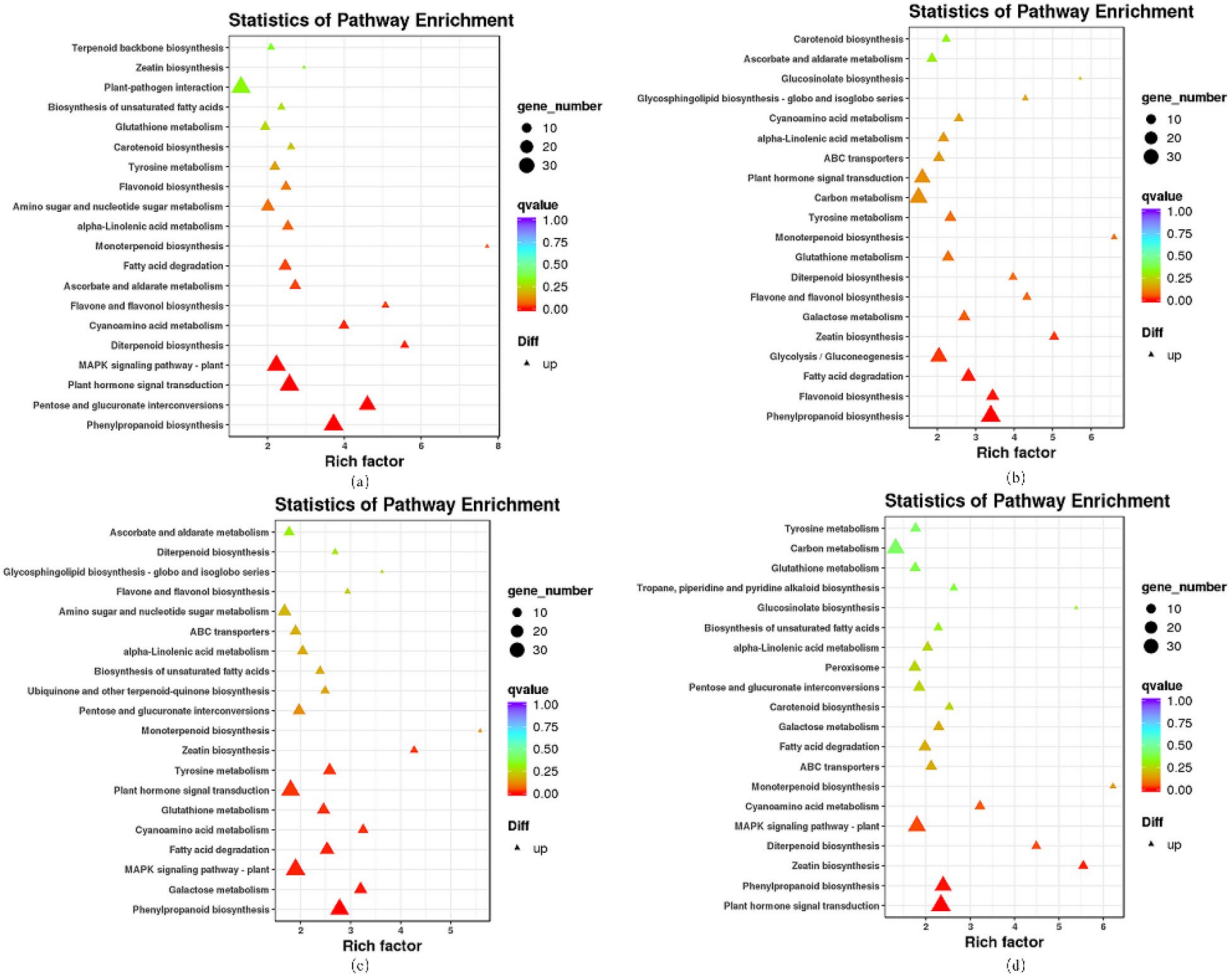
Statistics of KEGG enrichment for DEGs. (**a**) s0 vs. s1; (**b**) s0 vs. s2; (**c**) s0 vs. s3; (**d**) s0 vs. s4; Each circle in the figure represents a KEGG pathway, the Y-axis represents the pathway name, and the X-axis represents number of transcripts (circle size) enriched in the pathway, while the color code represents q-value and the size represents the gene number.

The KEGG database systematically analyses the metabolic pathways of gene products in cells and the function of these gene products (Fig. 4). Phenylpropanoid biosynthesis was the common metabolic pathways that were significantly enriched, and the presence of phenylpropanoids was related to the regulation of plant growth as well as to the defense against diseases. The pathways enriched to more genes in the s0 vs s1 stage were pentose and glucose interconversion, plant hormone signal transduction, MAPK signaling pathway-plant, MAPK signaling pathway is a signal transduction system that can mediate extracellular to intracellular signals through eukaryotic cells. Pathways enriched to a larger number of genes in the s0 vs s1 were flavonoid biosynthesis, fatty acid degradation, flavonoids contain a variety of polyphenol metabolites, which play different roles during plant development and adaptation^16^. The s0 vs s3 stages were mainly enriched in galactose metabolism, fatty acid degradation, cyanoamino metabolism, and the s0 vs s4 stages were significantly enriched in plant hormone signal transduction, zeatin biosynthesis.

### Screening of genes related to phytohormone signal transduction pathways

Auxin biosynthesis, transport and signal transduction play an important role in the occurrence and development of ARs; therefore, the expression of plant hormone-induced genes and related molecules has been widely studied. Based on GO and KEGG annotation analyses of all the DEGs, all four types of materials were found to be enriched in the plant hormone transduction pathway. Larch cutting propagation mostly belongs to the callus rooting type, so it can be considered that the production of calli is a very important stage. This process is not only a prerequisite for root primordium differentiation but also protects against the invasion of viruses and bacteria, restrains wound decay, preserves nutrients in the process of differentiation, and creates good conditions for stem cuttings to take root. Thus, we list the DEGs involved in hormone metabolism in the callus stage and AR induction stage (Table 2); 16 genes were upregulated, and 11 were downregulated. With the emergence of calli and the induction of ARs, key genes within the auxin, gibberellin, brassinosteroid, jasmonic and salicylic pathways were upregulated at all stages, such as *LAX1* (*Auxin transporter-like protein 1*), *GID2* (*Gibberellin insensitive dwarf 2*), *PYR1* (*Pyrabactin resistance 1*), *CRK1* (*Cysteine-rich receptor-like protein kinase 1*), *TIFY10B* (*Protein TIFY 10b*) and *TGA10* (*bZIP transcription factor TGA10*), while other genes showed consistently downregulated expression, such as *ARF4* (*Auxin response Factor 4*), *NSP2* (*Protein nodulation signalling pathway 2*), *BEH4(BES1/BZR1 homologue protein 4*) and *JAR1* (*JASMONATE RESISTANT 1*).

**Table 2:** Hormone-related common DEGs (log2FC) of larch in the two stages.

Hormone type	Gene number	s0 vs. s1	s0 vs. s2	Annotation	Database
Auxin	c141104.graph_c0	4.29	4.83	AUX1	KEGG/Swissprot
	c135836.graph_c0	− 1.29	− 1.78	ARF	KEGG/nr/Swissprot
Cytokinin	c158645.graph_c0	− 3.87	− 1.75	B-ARR	KEGG
	c147469.graph_c0	− 3.42	− 2.45	A-ARR	KEGG
	c157788.graph_c0	− 1.38	− 2.83	A-ARR	KEGG
	c158000.graph_c0	− 2.62	− 1.83	A-ARR	KEGG
Gibberellin	c155744.graph_c1	2.62	2.77	GID1	nr/Swissprot
	c121654.graph_c0	2.23	2.28	GID2	KEGG/Swissprot
	c126205.graph_c0	2.26	2.04	GID2	KEGG/Swissprot
	c152078.graph_c0	2.48	1.65	GID2	KEGG/Swissprot
	c129325.graph_c0	− 2.99	− 3.87	DELLA	KEGG
	c141744.graph_c0	− 3.83	− 3.42	TF	KEGG
	c158009.graph_c0	− 1.69	− 1.38	GID1	KEGG
Abscisic	c141172.graph_c0	5.35	6.13	PYR/PYL	KEGG
Brassinosteroid	c153133.graph_c1	3.38	4.74	BAK1	KEGG
	c158615.graph_c0	2.03	1.52	BAK1	KEGG
	c153361.graph_c0	− 1.82	− 2.16	BKI1	KEGG
	c150449.graph_c0	− 2.38	− 2.98	BZR1/2	KEGG
Jasmonic Acid	c142639.graph_c0	1.20	1.14	COI1	KEGG
	c154380.graph_c0	3.91	3.18	TIFY	Swissprot
	c158387.graph_c0	5.45	4.96	TIFY	Swissprot
	c156747.graph_c0	2.56	2.07	BHLH	Swissprot
	c149691.graph_c0	2.85	2.40	BHLH	Swissprot
	c147063.graph_c0	− 1.86	− 2.28	JAR1	KEGG
Salicylic acid	c156773.graph_c0	4.35	4.35	TGA	KEGG/Swissprot
	c147358.graph_c0	5.54	5.54	PR-1	KEGG
	c148550.graph_c0	4.02	4.02	PR-2	KEGG

### Screening of genes related to rooting

**Figure 5:**
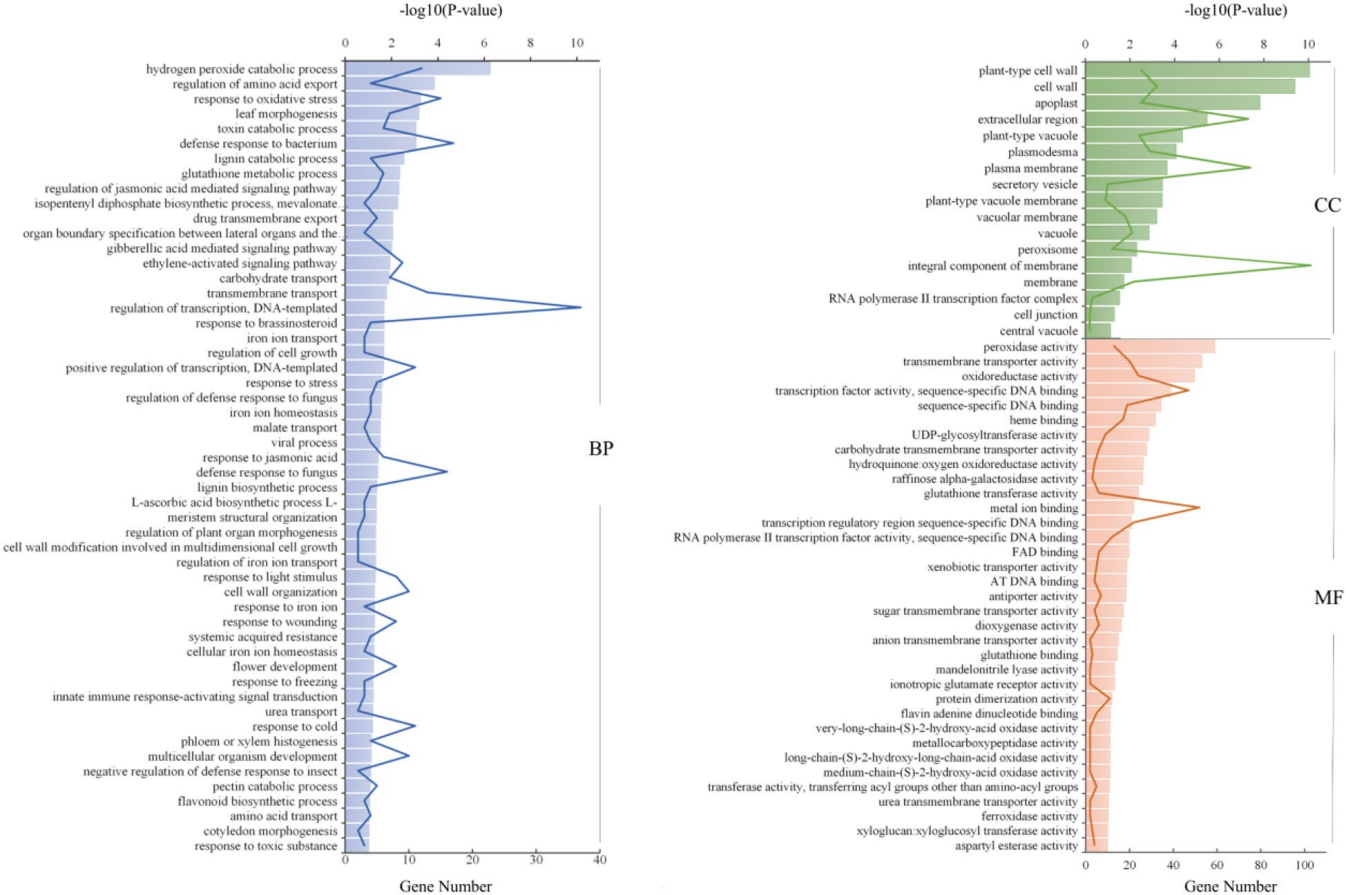
Statistics of GO annotation classification for the DEGs in s0 vs s1 and s0 vs s2. The bar chart represents the − log10 (P-value) of differential genes, line chart represents the number of Gene Ontology (GO) terms.

**Figure 6:**
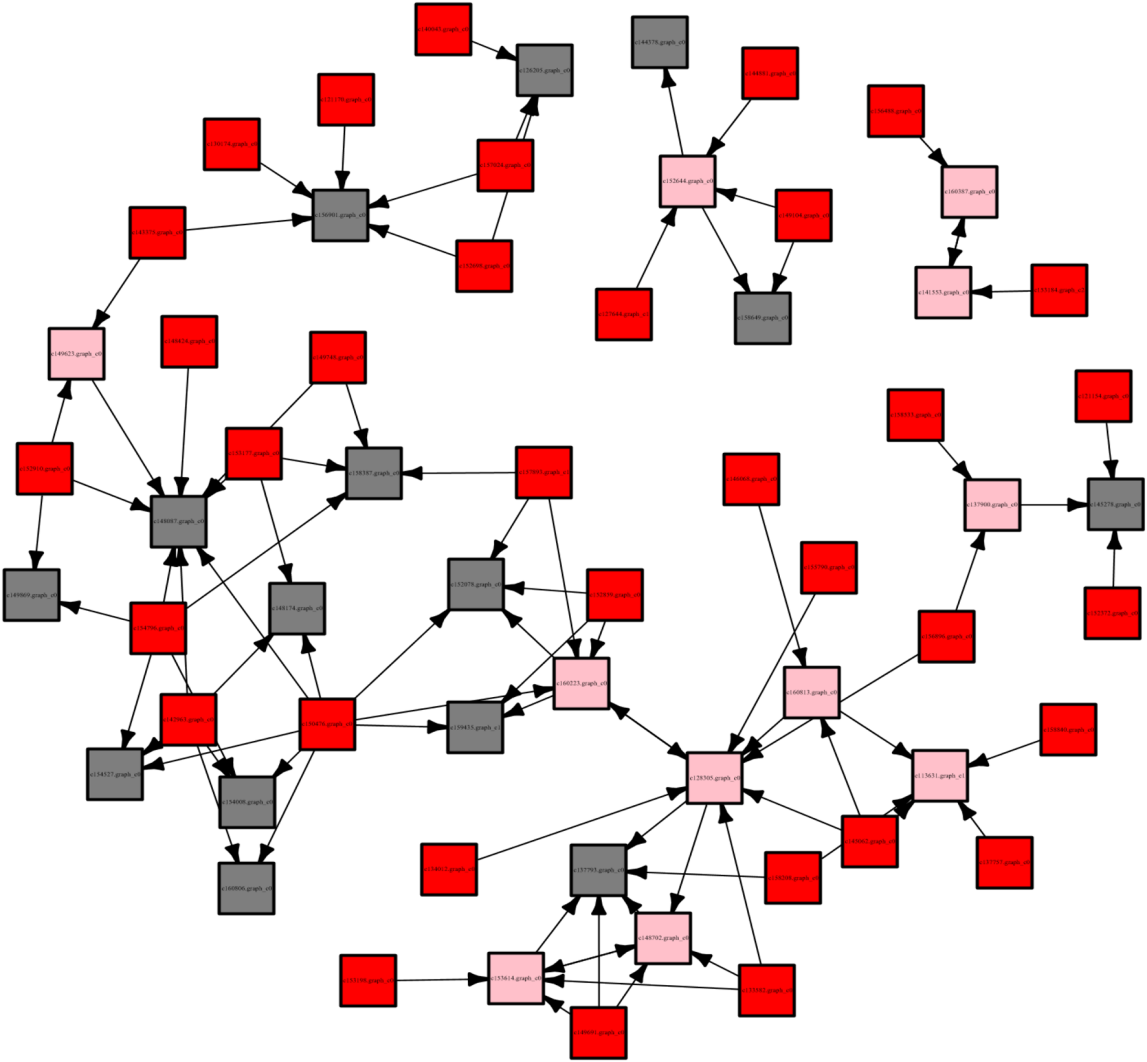
Gene regulatory network diagram (Pink represents the genes within the five pathways, red represents the genes outside the five pathways, and grey represents the functional proteins).

According to the results of POD, PPO IAAO indexes and GO analysis, I think s1 and s2 periods may be important periods in the rooting process of larch stem cuttings. To better identify the related genes that can promote rooting and formation, we selected the differential genes from the s0 vs. s1 and s0 vs. s2 comparisons for GO term enrichment analysis (Fig. 5) in metabolic pathways. Five metabolic pathways that may be related to rooting and formation were found: organ boundary specification between lateral organs and the meristem, gibberellic acid-mediated signalling pathway, ethylene-activated signalling pathway, regulation of plant organ morphogenesis, and cell wall modification involved in multidimensional cell growth. To identify the genes that are not in these five metabolic pathways but may regulate these metabolic pathways, we singled out all the differentially expressed transcription factors (TFs) and constructed a gene interaction network map (we deleted the differentially expressed TFs that were not found to regulate genes in the pathway) (Fig. 6). The higher the connectivity of the gene is, the more likely it is in the hub position in the network, which means that it has a more important role. After consulting the relevant literature, we identified 14 genes most likely related to rooting from the *LBD, AP2/ERF*, *MYB*, *NAC* and *bHLH* families and etc., then used them in the following experiments (Table 3).

**Table 3:** Core genes annotation.

Gene ID	TrEMBL_annotation	Species
c154796.graph_c0	AT-hook motif nuclear-localized protein 15-like	*Nelumbo nucifera*
c113631.graph_c1	R2R3MYB27	*Ginkgo biloba*
c160813.graph_c0	SNF2-related	*Macleaya cordata*
c133582.graph_c0	PPC domain-containing protein	*Nymphaea colorata*
c149623.graph_c0	LOB domain-containing protein	*Physcomitrium patens*
c160223.graph_c0	AP2/ERF domain-containing protein	*Corchorus capsularis*
c149691.graph_c0	BHLH domain-containing protein	*Picea sitchensis*
c157893.graph_c1	AT-hook motif nuclear-localized protein	*Macleaya cordata*
c153177.graph_c0	Trihelix transcription factor DF1	*Arabidopsis thaliana*
c150476.graph_c0	HTH myb-type domain-containing protein	*Picea sitchensis*
c153614.graph_c0	AP2/ERF domain-containing protein	*Picea sitchensis*
c128305.graph_c0	Aintegumenta-like transcription factor baby boom	*Larix gmelinii* var. *olgensis* × *Larix kaempferi*
c148702.graph_c0	AP2/ERF domain-containing protein	*Picea*
c142963.graph_c0	NAC transcription factor	*Pinus taeda*

### Quantification and validation of gene expression levels

**Figure 7:**
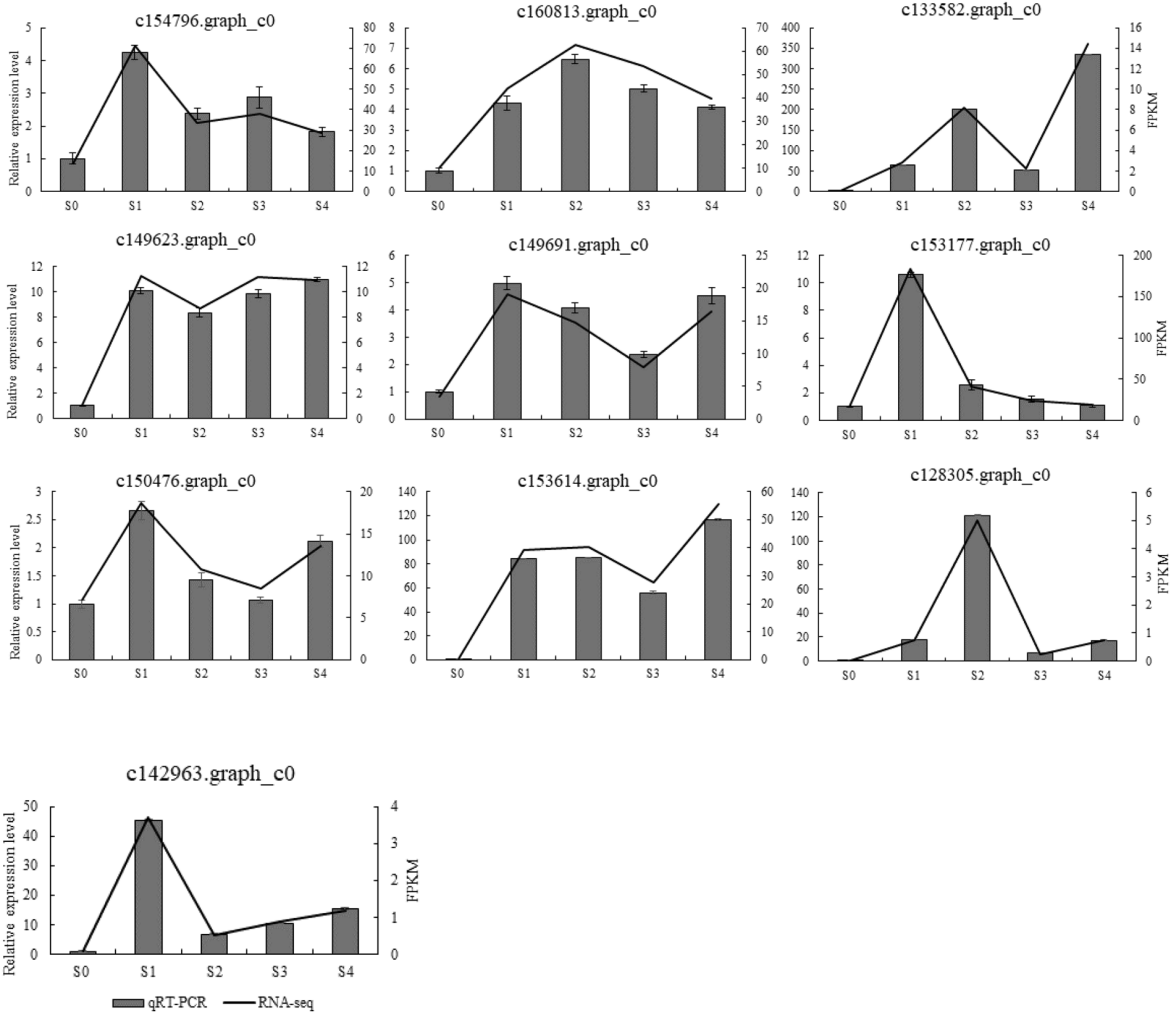
The relative expression levels of 10 DEGs based on RNA-Seq and qRT–PCR.

Ten rooting related genes were randomly selected for qRT–PCR to verify the reliability of the RNA-Seq data. The qRT–PCR assay results showed strong similarity with the RNA-Seq data (Fig. 7), which suggested that the RNA-Seq results were reliable, at the same time, it also verified the differential changes of these rooting related genes in the process of larch rooting.

## Discussion

Hybrid larch is the main afforestation tree species in northern China; it has important economic value, but the survival rate of stem cuttings of larch is low in alpine areas. It is also difficult for tissue culture plants to take root, and transgenic breeding is greatly restricted. It is well known that genome resources play an important role in forest genetics and breeding, but such resources in larch are scarce, which hinders the understanding of the molecular mechanism of AR formation. In this study, 43,246 unigenes sequences were generated by RNA-Seq. These sequences will help mine rooting-related genes in larch and further functional genome research.

Root formation in plants is closely related to the activity of oxidase, which plays an important role in this process. POD, PPO and IAAO are the three enzymes most closely related to AR formation. POD can catalyse the oxidation of various reductants related to hydrogen peroxide. It has been shown that POD participates in the decomposition of IAA and has the ability to catalyse the oxidative decarboxylation of IAA^17^. Many studies have shown that there are two peaks of POD activity during the induction and formation of AR from stem cuttings^18^. IAAO is a key enzyme in IAA catabolism that can degrade IAA and regulate IAA levels in plants. Wiesmann et al.^19^ concluded that low IAAO activity at the early stage of cutting was beneficial to the production of ARs. PPO can catalyse the metabolism of auxin and promote the origin and development of ARs. An important physiological function of the PPO enzyme is to catalyse phenols and IAA to form an "IAA phenolic acid complex", which is an auxiliary factor of rooting^20^. In this study, POD activity showed an "M"-shaped trend. Some products of POD action may be necessary auxiliary factors for the occurrence and development of ARs, and the two peaks are consistent with the results of previous studies. IAAO shows a down-up-down change pattern, as the activity decreased in the early stage of stem cutting but increased in the later stage of AR formation due to the weakening of IAA synthesis. The activity of PPO first increased and then peaked at the callus stage, and it gradually decreased with the induction of ARs, indicating that PPO could affect the induction and initiation of root primordia.

The plant hormone signal transduction pathway plays an important role in plant development^21^. Auxin, as one of the plant hormones, is widely used in plant reproduction to induce root formation during cutting propagation^22^. Auxin interacts with a series of other plant hormones through very complex crosstalk to regulate the levels and functions of these hormones, such as their biosynthesis, metabolism, transportation and signal transduction^23^. There were 25 auxin pathway genes involved in this study, including 3 *AUX1*, 6 *AUX/IAA* and 5 *ARF* genes. *AUX1* is distributed on the surface of the plasma membrane of the primary phloem of the root; it can promote the transport of IAA from the root to the root tip. Mutation of *AUX1* leads to a significant decrease in the content of auxin in the root tip. Due to the disturbance in IAA transport, development is delayed, and the number of lateral roots decreases^24^. External application of synthetic NAA can restore the lateral root phenotype of *AUX1*, which may be because the auxin content reaches a certain threshold to induce the expression of lateral root primordia. In this study, it was found that the expression of the larch gene c141104.graph_c0, which is homologous to the *AUX1* gene in *Arabidopsis thaliana*, was significantly upregulated in four groups of differentially expressed genes, indicating that *AUX1* may promote IAA transport in larch and affect AR development.

*AP2/ERF* family factors are divided into four main subfamilies: *DREB* (dehydration response element binding), *ERF* (ethylene response element binding protein), *AP2* (APETALA2) and *RAV* (related to *ABI3/VP*), with some soloists (few unclassified factors). *AtERF73/HRE1* is a member of the *AP2/ERF* family in *Arabidopsis thaliana*. Studies have shown that *HRE1 α* acts as a transcriptional activator in the nucleus and plays an important role in root development by regulating root meristem cell division^25^. Hirota et al.^26^ identified the *AP2/ERF* family gene *PUCHI* in *Arabidopsis thaliana*, which is involved in the initiation and development of lateral roots. *BBM* is a member of the *AP2* subfamily. Wang et al.^27^ cloned the *LkBBM1* and *LkBBM2* genes from hybrid larch (*Larix kaempferi* × *L. olgensis*) and transferred them into *Arabidopsis* and hybrid poplar (*Populus alba* × *P. glandulosa*). The results showed that overexpression of *LkBBM1* and *LkBBM2* in *Arabidopsis* significantly prolonged root growth, and overexpression in poplar significantly increased the number of ARs, so it is speculated that these two genes can regulate the development of ARs. In this study, six *AP2/ERF* family genes were found in the network map drawn by screening rooting-related genes, including a *BBM* gene, which is speculated to have a certain effect on the formation of ARs in larch stem cuttings. The lateral organ boundaries domain (*LBD*) gene family is a plant-specific TF family. *LBD* plays an important role in the regulation of plant growth, development and metabolism^28^. Previous studies have focused on the role of this gene family in the development of lateral organs, such as flowers, roots, stems, and leaves, and the formation of plant organs. Recently, it has been found that this gene family is closely related to plant physiological processes such as plant regeneration, pathogen response and abiotic stress signal response and participates in the molecular regulatory network of plant callus formation^29^. It was first found that *Arabidopsis AtLOB/AtASL4* was specifically expressed at the base of the adaxial end and the base of the lateral root of the lateral organs of *Arabidopsis*^28^. In addition, it has been reported that *LBD16*, *LBD29* and *LBD18* are direct or indirect targets for *ARF7* and *ARF19* to regulate lateral root formation^30, 31^. *AtLBD16* and *AtLBD29*, as well as *AtLBD17* and *AtLBD18*, are key regulators in the process of lateral root formation in *Arabidopsis*. Four *LBD* genes were rapidly upregulated in the early stage of callus induction of root and bud explants. Further analysis showed that *LBD* TFs were key regulators guiding callus formation in an in vitro plant regeneration system^32^. In this study, four *LBD* genes were found in the differentially expressed genes based on the transcriptome, and the expression of these four genes was upregulated in all stages, so we speculated that this gene may participate in the formation of calli and promote root formation in the process of larch stem cuttings.

## Conclusions

The consistency of the dynamic changes in POD, PPO and IAAO activity in the rooting process of larch stem cuttings showed that the activities of these three enzymes were closely related to the development of adventitious roots of larch stem cuttings. The s1 stage was an important period for the development of adventitious roots of larch stem cuttings. Through transcriptome analysis of four stages of adventitious root development of hybrid larch, a total of 43,246 unigenes were obtained. According to GO analysis and KEGG enrichment pathway analysis, we found that plant hormone signal transduction played an important role in the rooting process of larch stem cuttings, and 9 plant hormones were involved in the plant hormone signal transduction pathway, a larch gene c141104.graph_c0 that is homologous to the Arabidopsis *AUX1* was found to be significantly up-regulated. We suggest that *AUX1* may promote IAA transport in larch, thus affecting adventitious root development Five metabolic pathways that may be related to rooting were found in the GO pathway enrichment of s0 vs. s1 and s0 vs. s2, and 14 genes that may be related to rooting were identified for verification in subsequent experiments. This study will provide a reference for improving the asexual reproduction rate of larch.

## Materials and methods

### Plant materials

**Figure 8:**
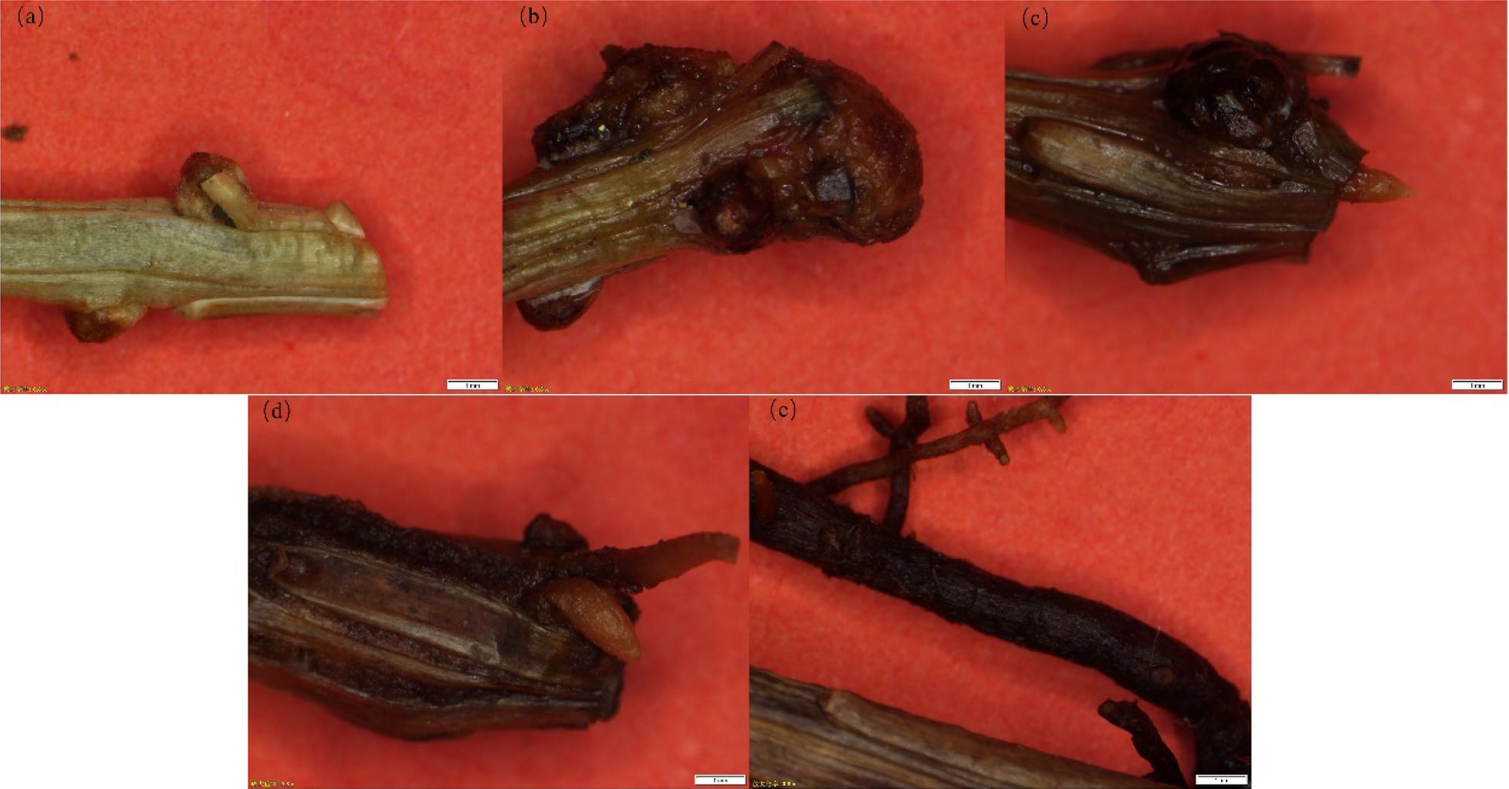
Rooting initiation stage (**a**), callus formation stage (**b**), adventitious root induction stage (**c**, **d**) and adventitious root growth stage (**e**).

Three 8-year-old clones (*Larix gmelinii7* × *Larix kaempferi77-2*, *Larix kaempferi5* × *Larix gmelinii9*, and *Larix gmelinii9* × *Larix kaempferi76-2*) from the hybrid larch seed orchard of the National Larch Improved Seed Base in Linkou County (45°24′45.49ʺ N, 130°32′55.10ʺ E), Heilongjiang Province, China, were used for cutting propagation, and the quicksets were 15 cm long and soaked in 200 ppm IBA solution for 30 min. Four stages of rooting after cutting were selected: the rooting initiation stage, callus formation stage, AR induction stage and AR growth stage (Fig. 8). Because the period of AR induction is longer in the process of rooting, it is divided into two sampling intervals of approximately 10 days. Sixty plants were selected in each stage, which were named s0 (stage 0), s1 (stage 1), s2 (stage 2), s3 (stage 3) and s4 (stage 4). A portion from the bottom of each cut stem was taken as the research object. After setting up three biological replicates, the base samples were quickly frozen in liquid nitrogen and stored in the refrigerator at – 80 °C.

### Determination of physiological indices

The root samples of the above five stages were collected to determine the activities of POD, PPO and IAAO using the Suzhou Grace Biotechnology Company Kit (spectrophotometer method) according to the instruction manual.

### RNA extraction and cDNA library construction

A TRIZOL kit (Invitrogen, USA) was used to extract RNA from 15 samples separately across the s0–s4 stages, with 3 biological repeats. After the total RNA was detected by a spectrophotometer and agarose gel electrophoresis, the mRNA was separated and fragmented, and the double-stranded cDNA was synthesized by reverse transcription. Then, the sequencing library was constructed by 5′ terminal repair, 3′ terminal addition, ligation, and enrichment. The constructed library was sent to Biomarker Technologies in Beijing, China, and the cDNA library was sequenced using the Illumina HiSeq 2000 platform.

### Quality control and transcriptome assembly

The cDNA library was sequenced on an Illumina HiSeq 2000 platform to produce a large number of high-quality reads. The reads or bases produced by the sequencing platform were called raw data, and clean data (clean reads) were obtained by removing reads containing adapters, reads containing poly-N sequences and low-quality reads from the raw data. After obtaining high-quality sequencing data, sequence assembly was performed using Trinity^33^.

### Screening of differentially expressed genes

For the experiments with biological repetition, DESeq2^34^ was used to analyse the differential expression among the sample groups. In the process of differential expression analysis, the generally accepted and effective Benjamini–Hochberg method was used to correct the significant P-value obtained by the original hypothesis test, and finally, the corrected P-value, FDR < 0.01 and FoldChange > 2, was used as the key index for differentially expressed gene screening.

### Functional annotation, classification, metabolic pathway analysis and genetic regulatory networks of unigenes

The unigene sequences were compared with the NR, Swiss-Prot, COG, KOG, eggNOG4.5 and KEGG databases by DIAMOND software, and the KEGG pathways related to the unigenes were obtained by KOBAS. InterProScan uses the integrated InterPro database to analyse the GO results of queried genes. After initially screening the amino acid sequences of the unigenes, HMMER software was used to compare the Pfam database results and obtain the annotation information and functional classification of unigenes. We used GENIE3 software^35^ to build a genetic regulatory network.

### Quantitative real‑time PCR analysis

Based on the cDNA library of five stages constructed in the first step described above, 10 differentially expressed genes (DEGs) were randomly selected. Primer5 software was used to design quantitative primers (Table 4). An ABI7500 fluorescent qRT–PCR instrument was used to determines the dissolution curve according to standard procedures, with the following reaction conditions: 94 °C for 30 s, followed by 40–45 cycles of 94 °C for 5 s, 58 °C for 15 s, and 72 °C for 10 s. The differences in the three CT values were all less than 1. Microsoft Excel 2016 was used for data analysis, and GraphPad Prism 5 software was used for graphing. The internal reference gene of *Larix olgensis* is MF278617 on NCBI. The s0 stage sample was used as the control. The gene relative expression levels were determined by 2^−ΔΔCT^as expressed.

**Table 4:** The primers used for real-time RT–PCR.

Gene ID	Forward primers (5′–3′)	Reverse primers (5′–3′)
c149623.graph_c0	ATATTCGGAGCGAGCAACG	CTCCCACGCACCCATAGAC
c153177.graph_c0	TAGCCGTCCAGTGCAGAATC	TTAGGGTTCTTAAGCAAAGTTGC
c154796.graph_c0	GTCAGGGGAAAAGGAGAACC	GCCCAATTTCATCCCCTAC
c142963.graph_c0	TTCCAAAAGGATTGACCTAGATG	CTTTCCAGAAGCCAGCCAC
c150476.graph_c0	GTATCAGATGAAGAAGCCCACC	AGCTCTGCAGTCCATCTCAAAC
c149691.graph_c0	ATTGTATCTGCAGGGCTTTATG	TGATCCATTGCGATGACTTC
c133582.graph_c0	TCACCAACTTGACCCTCCG	GCTTCCTCCCATTACCTGC
c160813.graph_c0	GATCTTGGCCTCGTTGCCC	ACATCTTTCTCGCCATTTC
c128305.graph_c0	GGCTTAAAAGTAGATAGTTGCCG	ACCTTAGCTCCTACTTCGCTCAC
c153614.graph_c0	TTGCTGGATGTGGAGGACG	CTGGTGGTGGTAATGGCTTG

### Statement

Our experimental research and field studies on plants are comply with comply with the IUCN Policy Statement on Research Involving Species at Risk of Extinction and the Convention on the Trade in Endangered Species of Wild Fauna and Flora.

All methods were carried out in accordance with relevant guidelines.

## Data Availability

The Illumina raw data were submitted to the General Services Administration (GSA) at CNCB accession number CRA006979 under the following link: https://ngdc.cncb.ac.cn/search/?dbId=&q=CRA006979.
